# A matched case–control study of preterm birth in one hospital in Beijing, China

**DOI:** 10.1186/1742-4755-12-1

**Published:** 2015-01-05

**Authors:** AiQun Huang, Xi Jin, XiaoHong Liu, SuHong Gao

**Affiliations:** National Center for Women’s and Children’s Health, China CDC, Beiyuan Road, Chaoyang District, Beijing, China; Haidian Maternal and Child Health Care Hospital, Haidian Nan Road, Haidian District, Beijing, China

**Keywords:** Preterm birth, Case–control study, Risk factors

## Abstract

**Background:**

Preterm birth is an unresolved global health issue. The etiologies of preterm birth are complex and multifactorial. To examine risk factors related to preterm birth, a matched case–control study was conducted in a hospital in Beijing, China where little data on preterm birth have been published in the scientific literature.

**Methods:**

A 1:1 matched case–control study was conducted in 172 pairs of women with preterm birth (case group) and term delivery (control group). Eligible subjects were interviewed in person by well-trained investigators using a questionnaire. Information on obstetric diagnosis and newborns were abstracted from inpatients’ medical records. Univariate and multivariate conditional logistic regression models were used to measure the associations between related factors and preterm birth.

**Results:**

Univariate analysis showed that 6 of 12 factors were associated with preterm birth. Multivariate results showed that gestational hypertension (OR = 7.76), low gestational weight gain (OR = 3.02), frequent prenatal care (OR = 0.16), balanced diet (OR = 0.36), and high gestational weight gain (OR = 0.41) were associated with preterm birth.

**Conclusion:**

This study provides information on preterm birth in Beijing, China, and it also lends support to existing evidence about the role of maternal nutritional status, prenatal care and gestational hypertension as risk factors for preterm birth.

## Background

Preterm birth is defined as childbirth occurring at less than 37 completed weeks of gestation, or fewer than 259 days since the first day of a woman’s last menstrual period. Preterm birth is an unresolved global health issue. This condition is also the largest contributor to neonatal mortality and the second largest contributor to all under-5 mortalities [1, 2]. Besides the contribution of preterm birth to mortality, preterm birth has an increased risk of chronic disease in adulthood, such as hypertension [3, 4], type 2 diabetes [5], low exercise capacity [6], and even early death [7]. The Global Burden of Disease study in 2010 showed that, for all ages and causes, complications from preterm births make up the seventh cause of disability-adjusted life years in developing countries and the 35th cause in developed countries [8]. A national, regional, and worldwide estimate of the preterm birth rate for 2010 from 184 countries was 11.1%, amounting to approximately 14.9 million preterm births [9]. Although the preterm birth rate in China is 7.1%, the rank for number of preterm births is the second (approximately 1.2 million in 2010) in the world.

There are many maternal or fetal characteristics that are associated with preterm birth, including maternal demographic characteristics, nutritional status, history of pregnancy, present characteristics of pregnancy, psychological characteristics, adverse behavior, infection, uterine contractions and cervical length, and biological and genetic markers [10, 11]. Despite considerable research in developed countries, little is known regarding the causes of preterm birth in developing countries.

Research on risk factors related to preterm birth in China, where there is a high number of preterm births annually, is scarce. As the capital of China, Beijing has special characteristics in economics, education, culture, and health care. Only one study has reported risk factors for preterm birth in Beijing [12]. Therefore, to examine risk factors related to spontaneous preterm birth, we conducted a matched case–control study in a hospital in Beijing, China, a country and city for which little data on preterm birth has been previously published or is publicly known.

## Methods

This study was designed as a 1:1 matched case–control study.

### Study subjects

This study was conducted in Beijing Haidian Maternal and Child Health Care Hospital (Haidian MCH) during July 2010 to December 2011. Haidian MCH is one of the largest maternity hospitals in Beijing urban area. Haidian MCH has approximately 12,000 annual deliveries, accounting for approximately 8% of the total delivery number in Beijing. Almost all of the population that is served by Haidian MCH is from urban areas. In Beijing, the general model of maternity health care is that once a woman selects a hospital for her delivery at the beginning of pregnancy, her entire maternity health care service from pregnancy to delivery will be performed at this hospital. Therefore, almost all of our subjects received their maternity health care service in Haidian MCH from the beginning of pregnancy.

Cases were defined as women who delivered a live singleton newborn between 28 and 36 weeks of gestation, and birth weight was less than 2500 g. Controls were women who delivered a live singleton newborn at term (37–41 weeks of gestation) with a normal birth weight (3000–4000 g). An eligible control was matched to a case by delivery date (within 3 days), maternal age (within 3 years), and newborn’s sex. Exclusion criteria were as follows: (1) women refused to participate; (2) women had an acute or serious chronic disease; (3) preterm birth due to medical intervention because of pregnancy complications; and (4) the newborn had a birth defect.

### Data collection procedure

After obtaining informed consent, the eligible subjects were interviewed in person by well-trained investigators using a questionnaire. Questionnaire information included demographic characteristics, history of disease, reproductive history, lifestyle and nutritional status during pregnancy, and reproductive tract infection during pregnancy. After the questionnaire-based interview, information on each subject’s obstetric diagnosis during pregnancy and delivery (e.g., gestational hypertension and diabetes mellitus), and their newborns (e.g., Apgar score and birth weight) was abstracted from inpatients’ medical records by trained investigators.

### Variables used in analysis

Newborns were weighed by nurses within 1 hour of delivery on a calibrated electronic balance. Gestational age at delivery was calculated according to the confirmed last menstrual period. If women who had a poor recall of the last menstrual period, the earliest ultrasonographic measurement was used to estimate the gestational age.

Because most of subjects were from the urban area of Beijing, China, they generally had a higher educational level than other areas. Therefore, education was categorized as “lower education” (including illiteracy, elementary, and middle school) and “higher education” (including university or graduate education).

With regard to the family conditions of subjects, family income was categorized as < 1000 dollars and ≥ 1000 dollars per month. Average living space was classified as < 20 m^2^ and ≥ 20 m^2^ per person.

According to the national guideline of maternity health care and the fact that most pregnant women in large cities in China have frequent prenatal examinations during pregnancy, in this study, prenatal care visits were defined as < eight times and ≥ eight times. Other maternal health factors were all categorized according to subjects’ answers to the questionnaires. The diagnoses of gestational hypertension and diabetes mellitus were abstracted from inpatients’ medical records.

Body mass index (BMI) was defined as body weight divided by the square of height in kg/m^2^. Pre-pregnancy BMI was classified into three categories [13]: < 18.5, 18.5–23.9, and ≥ 24. Because there are no consistent criteria of gestational weight gain for Chinese women, we ranked gestational weight gain into three groups: low, normal, and high.

### Ethics statement

All of the study protocols were reviewed and approved by the Ethics Committee for Human Subjects Studies of the National Center for Women and Children’s Health, Chinese Center for Disease Control and Prevention.

### Statistical methods

All analyses were performed with the statistical package SAS version 8.2 (SAS Institute Inc., Cary, NC, USA). The paired-sample t-test was used for comparison of continuous variables. For categorical variables, univariate conditional logistic regression was used to determine the odds ratio (OR). Adjusted ORs for preterm birth were determined by multiple conditional logistic regression. A probability value of P < 0.05 was considered statistically significant, and ORs and 95% confidence intervals (95% CIs) are presented.

## Results

A total of 172 case–control pairs were analyzed in this study. Characteristics of the subjects are shown in Table 1. In the preterm birth group, the mean gestational week and birth weight were 33.9 ± 1.9 weeks and 2022.3 ± 338.9 g, respectively. Maternal age, husband’s age, and history of delivery were not significantly different between cases and controls. None of the subjects had a past medical history, history of preterm delivery, or exposure to smoking, drinking, radiation, and chemical substances. With regard to the newborns, 97 pairs were girls and 75 pairs were boys.

**Table 1:** **Characteristics of the subjects**

	Control	Case
Gestational weeks	39.1 ± 1.0	33.9 ± 1.9
newborn’s birth weight (g)	3416.0 ± 241.4	2022.3 ± 338.9
Maternal age (years)	29.1 ± 3.3	29.2 ± 3.8
Husband’s age (years)	30.9 ± 4.1	31.0 ± 4.6
History of delivery		
no	153 (88.9)	153 (88.9)
yes	19 (11.1)	19 (11.1)

History of delivery was showed as n (%), others were showed as Mean ± sd.

### Univariate analysis

#### Socio-economic status

As shown in Table 2, preterm birth occurred more frequently in women who had less family living space (OR = 1.90, 95% CI 1.09–3.30).

**Table 2:** **Univariate analysis of socio-economic status and preterm birth**

	Control		Case		OR	95% CI	P
	n	%	n	%			
Education							
lower education	31	18.0	39	22.7	1		
higher education	141	82.0	133	77.3	0.72	0.41-1.27	0.260
Family income(dollars, per month)							
≥1000	78	45.3	70	40.7	1		
<1000	94	54.7	102	59.3	1.31	0.79-2.18	0.303
Family living space (m^2^, per person)							
≥20	123	71.5	106	61.6	1		
<20	49	28.5	66	38.4	1.90	1.09-3.30	0.024

#### Maternal health during pregnancy

As shown in Table 3, women with frequent prenatal care visits (≥8 times) were less likely to have preterm birth than those with less than eight visits during pregnancy (OR = 0.20, 95% CI 0.10–0.40), while gestational hypertension was a risk factor of preterm birth (OR = 5.50, 95% CI 2.59–11.68).

**Table 3:** **Univariate analysis of maternal health during pregnancy and preterm birth**

	Control		Case		OR	95% CI	P
	n	%	n	%			
Times of prenatal care visits							
<8	17	9.9	54	31.4	1		
≥8	155	90.1	118	68.6	0.20	0.10-0.40	<0.001
Anxiety during pregnancy							
no	122	70.9	116	67.4	1		
yes	50	29.1	56	32.6	1.18	0.74-1.88	0.480
Reproductive tract infection during pregnancy							
no	124	72.1	110	64.0	1		
yes	48	27.9	62	36.0	1.52	0.93-2.47	0.092
Gestational Hypertension							
no	163	94.8	127	73.8	1		
yes	9	5.2	45	26.2	5.50	2.59-11.68	<0.001
Gestational diabetes mellitus							
no	155	90.1	146	84.9	1		
yes	17	9.9	26	15.1	1.64	0.85-3.19	0.143

#### Maternal nutritional status

Table 4 shows that balanced diet was associated with a decreased risk of preterm birth (OR = 0.43, 95% CI 0.22–0.84). Women with a pre-pregnancy BMI ≥ 24 had a higher risk of preterm birth (OR = 2.15, 95% CI 1.14–4.06) than those with a BMI of 18.5–23.9. Compared with the women whose gestational weight gain was normal, the women who had low gestational weight gain had an increased risk of preterm birth (OR = 3.50, 95% CI 1.84–6.66), while the women who had a high gestational weight gain had a decreased risk of preterm birth (OR = 0.42, 95% CI 0.23–0.76).

**Table 4:** **Univariate analysis of maternal nutritional status and preterm birth**

	Control		Case		OR	95% CI	P
	n	%	n	%			
Balanced diet							
no	18	10.5	34	19.8	1		
yes	154	89.5	138	80.2	0.43	0.22-0.84	0.014
Supplement folic acid during pregnancy							
<3 months	51	29.7	65	37.8	1		
≥3 months	121	70.3	107	62.2	0.64	0.39-1.06	0.083
Pre-pregnancy BMI (kg/m^2^)	20.8 ± 2.5		21.4 ± 2.9				
<18.5	30	17.4	16	9.3	0.55	0.28-1.08	0.083
18.5-23.9	122	70.9	117	68.0	1		
≥24	20	11.6	39	22.7	2.15	1.14-4.06	0.018
Gestational weight gain (kg)	17.0 ± 4.6		13.3 ± 5.0				
normal growth (15.2 ± 1.1)	60	34.9	55	32.0	1		
low growth (10.6 ± 2.2)	31	18.0	86	50.0	3.50	1.84-6.66	<0.001
high growth (21.4 ± 3.5)	81	47.1	31	18.0	0.42	0.23-0.76	0.004

Pre-pregnancy BMI (kg/m^2^) and gestational weight gain (kg) were also showed as Mean ± sd.

### Multivariate analysis

The results of multiple conditional logistic regression analysis are shown in Table 5. Significant risk factors for preterm birth were gestational hypertension (OR = 7.69, 95% CI 2.77-21.32) and low gestational weight gain (OR = 3.02, 95% CI 1.36-6.68). However, women who had ≥ eight prenatal care visits (OR = 0.16, 95% CI 0.06-0.44), a balanced diet (OR = 0.36, 95% CI 0.14-0.91) and high gestational weight gain (OR = 0.41, 95% CI 0.18-0.96) were less likely to have preterm birth.

**Table 5:** **Multivariate analysis for related factors of preterm birth**

Related factors	β	Walds***χ*** ^2^	OR	95% CI	P
University or graduate education	0.097	0.050	1.10	0.47-2.57	0.823
Family income <1000 dollars per month	0.386	0.875	1.47	0.66-3.31	0.350
Family living space <20 m^2^ per person	−0.234	0.254	0.79	0.32-1.97	0.614
Prenatal care visits ≥ 8 times	−1.852	12.458	0.16	0.06-0.44	<0.001
Anxiety during pregnancy	0.482	1.784	1.62	0.80-3.28	0.182
Reproductive tract infection during pregnancy	0.602	2.758	1.83	0.90-3.72	0.097
Gestational hypertension	2.040	15.349	7.69	2.77-21.32	<0.001
Gestational diabetes mellitus	0.2110	0.180	1.24	0.47-3.27	0.671
Balanced diet	−1.023	4.717	0.36	0.14-0.91	0.030
Supplement folic acid ≥3 months during pregnancy	−0.162	0.197	0.85	0.42-1.74	0.657
Pre-pregnancy BMI <18.5 kg/m^2^	0.145	0.086	1.16	0.44-3.05	0.769
Pre-pregnancy BMI ≥24 kg/m^2^	0.362	0.632	1.44	0.59-3.50	0.427
Low growth weight gain	1.104	7.402	3.02	1.36-6.68	0.007
High growth weight gain	−0.886	4.226	0.41	0.18-0.96	0.040

## Discussion

Preterm birth is a major public health issue and the most important clinical problem in obstetrics and neonatal medicine. The recent published global action report on preterm birth clearly outlines that prevention of preterm birth must be accelerated through family planning, improved quality of care before and during pregnancy and between pregnancies, and strategic investments in research and innovation [14, 15].

The etiology of preterm birth is still largely unknown. There are likely to be multifactorial etiologies of preterm birth, each with a distinct biological pathway. The etiologies differ according to gestational age, ethnicity, and characteristics unique to each population. Each pathway to preterm birth may be also influenced by gene-environment interactions and genetic variability [10, 11]. Recent research has highlighted that there is a need for clarity in the definition of preterm birth, including gestational age and distinguishing between spontaneous preterm birth (which occurs naturally because of preterm labor or premature rupture of the fetal membranes) and indicated preterm birth (in which labor is initiated by medical intervention because of pregnancy complications) [16]. In our study, cases were spontaneous preterm birth and birth weight was less than 2500 g. Therefore, to some extent, this study can minimize the heterogeneity of etiology of preterm birth.

Maternal nutritional status before and during pregnancy is important for the health of women and their growing fetus. In the present study, the definition of a balanced diet was simple. A balanced diet before and during pregnancy was associated with a decreased risk of preterm birth, which is similar to another study in Beijing [12]. With regard to BMI, the association of preterm birth with low BMI was consistent [17, 18], but was inconsistent with overweight or obese, as previously shown [19]. In our study, pre-pregnancy BMI ≥ 24 was associated with preterm birth in univariate analysis, but was not statistically significant after adjusting for other factors.

With regard to the effect of gestational weight gain on preterm birth, we found that low weight gain was a risk factor of preterm birth, whereas high weight gain was a protective factor of preterm birth compared with normal gestational weight gain. This finding is consistent with other studies [20, 21]. Normal growth and development of a fetus results in a normal pregnancy, and this depends on several factors. One of the factors is maternal nutritional status before and during gestation, and this is related to appropriate transport of metabolic substrates to supply energy requirements of the growing fetus. Disorder of this equilibrium can affect the outcome of pregnancy in different ways [21]. The Institute of Medicine has recommended that weight gain should be stratified by BMI during pregnancy [22]. However, Chinese-specific recommendations of the range in weight gain have not been reported. Therefore, the relationship between preterm birth and gestational weight gain stratified by pre-pregnancy BMI needs to be studied further in China.

In our study, women with frequent prenatal care were less likely to have preterm birth, which is compatible with previous studies [23, 24]. Effective and available prenatal care can detect and cope with risk factors of preterm birth and other complications of pregnancy. In China, especially in urban areas, pregnant women can have more than eight prenatal care visits during pregnancy. More attention needs to be paid to the effectiveness and quality of each prenatal care visit, especially for women with risk factors of preterm birth or other complications.

Similar to previous studies [25, 26], our study also showed that gestational hypertension increased the risk of preterm birth. The mechanism for this finding may be related to reduced placental blood flow, which should affect the exchange of nutrients and oxygen between the mother and fetus. This would result in decreased fetal growth and increase the risk of abnormal pregnant outcomes, such as intrauterine growth restriction, low birth weight, and preterm birth.

In our study, some related factors, such as gestational diabetes mellitus, reproductive tract infection during pregnancy, education, family income, and living space, were not significantly associated with preterm birth. Although the etiology of preterm birth remains unclear, the different habits and demographic characteristics of the population may in part explain the discrepancies with previous studies.

There are some limitations in this study. First, our findings may be limited by recall bias during the interview for the questionnaire. Second, in our study, to enable more homogeneity of spontaneous preterm infants, cases were defined as women who delivered a live singleton newborn between 28 and 36 weeks of gestation and birth weight was less than 2500 g. Therefore, children who were born before 28 weeks of gestation or weighed more than 2500 g were excluded, even though technically, these newborns were preterm. Finally, because of the limited sample size, our study did not classify preterm birth by sub-categories according to different gestational ages. Therefore, factors of different sub-categories that affect preterm birth need to be further studied.

## Conclusions

This study lends support to existing evidence regarding the role of maternal nutritional status before and during pregnancy, prenatal care, and gestational hypertension as risk factors for preterm birth. Although our findings may not be generalized beyond a hospital population and require further exploration and validation, they should serve as the first step in identifying risk factors in Beijing, China.
